# Traumatic Neurasthenia

**Published:** 1902-03-08

**Authors:** Arthur Maude

**Affiliations:** Medical Officer of Charing Cross Hospital Convalescent Home


### Hospital Clinics and Medical Progress.

### TRAUMATIC NEURASTHENIA.

By Arthur Maude, M.R.C.S., L.R.C.P., Medical Officer of Charing Cross Hospital Convalescent Home.

The conditions formerly known as "railway spine," "railway brain," and "coal-miner's neurosis," are all grouped now under the better title of *Traumatic Neurasthenia.* Such conditions came formerly under the cognisance of a very limited number of surgeons. Bicycles have altered all this, and few practitioners can expect to pass a year without being called upon to manage one or more of these difficult cases. This is not surprising, because, as Charcot pointed out long ago, neurasthenia is produced rather by the suddenness of an accident than by the degree of physical damage, and no class of accident produces such sudden degrees of shock with such slight damage of tissue as those sustained on bicycles. No accidents are so sudden as those on bicycles. A healthy man may be precipitated from his machine by a jolt, reach the ground and become unconscious from concussion before his sensorium has realised that his seat is disturbed. In horse accidents, railway collisions, or mountain falls, the few seconds of elapse, between the realisation of impending danger and the shock itself, produce a lower rate of neurasthenic sequels in the victim than is the case in bicycle accidents. This is only one factor; another is the previous nervous conditions of the subjects of accident. The railway engineman, the coal miner, the hunting man are all people in whom we should find a uniform high potentiality of nerve discharge; unless they are alcoholic they are unlikely to become neurasthenic, while the modern bicycle rider may come of any grade of nervous power.

We must be allowed to beg the question that there is a symptom complex recognisable as neurasthenia; that there is a well-known group of conditions of nervous failure, unfortunately too common, which cannot be classified as "neuralgia, cephalic sensations, hysteria, or hypochondriasis." The general condition is now so well known that we do not propose to survey neurasthenia in general, but to discuss the points of contact and variation between nervous exhaustion of idiopathic and traumatic production. It is obvious in starting that it is frequently impossible to separate the causation completely. An accident which in a robust nervous system will produce no effect beyond temporary shock, perhaps even profound concussion with complete recovery, will in the subject who has been previously submitted to constant overwork, lack of holiday, and continued worry, determine a profound and perhaps long-abiding state of neurasthenia. In most cases "the symptoms of traumatic neurasthenia are chronic in character and development, and are exactly similar to those of neurasthenia induced not by traumatism, but by worry and overwork" (Victor Horsley). To this we subscribe with one reservation, that in cases in which traumatism has been suddenly superadded to other causes the onset of recognisable neurasthenia is very sudden and acute. Mr. Horsley claims that when the symptoms immediately follow the accident "they are undoubtedly mixed with genuine symptoms of concussion," and that therefore some organic though minute lesions have possibly been effected, which, in the absence of microscopic post-mortem investigation, cannot be gainsaid, especially in cases of great acuteness. But it is certainly in the writer's experience that bicycle spills, and blows on the vertex, will produce signs of mild neurasthenia in a few hours, without any unconsciousness or disturbance of cerebration, in persons probably the subjects of a latent, previously existing mild form of neurasthenia. The accidents may be very slight, a blow in bringing back a "driver" in addressing a golf ball, by which a bystander was struck; a blow on the temporal region from a handle in a bicycle fall; striking the vertex in stooping under the bar of a loose box are examples in the writer's practice which have produced lasting conditions of neurasthenia in the subjects of existing mild neurasthenic states.

Traumatic neurasthenia may exceptionally be the most acute form of the neurasthenic state. The most striking feature of this condition is a hyperpyrexia which under other circumstances of production is quite incompatible with life. A temperature of 108°, caused by the few conditions which produce such a temperature, was regarded a few years ago as mortal. A temperature of 111° was almost unknown, and usually only recorded in rare cases of acute rheumatism, enteric, or pneumonia, and within an hour or two hours of death. Horsley has recorded cases of such hyperpyrexia which have recovered; one in which the slightest excitation would send the temperature up to 107°.

The writer has seen a case of chronic, profound neurasthenia in which sun exposure in England produced absolute coma, lasting many hours, with a temperature of 106°; ice-packing and rubbing with blocks of ice brought it down readily, and within a few days the patient seemed as well as before the attack The case merely exemplifies the extreme instability of the heat-producing centres in neurasthenics, and the consequent attention that must be paid to the history of such patients in estimating the value of their temperatures in ordinary pyrexial diseases, particularly influenza.

The ease with which these extreme high temperatures can be reduced is significant, if not pathognomonic. Horsley says, "the application of cold brings the temperature down almost as quickly as it rose." It is quite otherwise in the temperatures of typhoid or rheumatism.

The type of injury which produces traumatic neurasthenia has been classified by Horsley: the commonest form is shock directly applied to the central nervous system, whether brain or cord; in addition he places injuries to blood vessels, ruptures of muscles or tendons, and injuries of bones. We might add, the extensive skin destruction which may accompany bicycle accidents on hard roads. Probably the factor in all the injuries of skeletal tissues is the extensive irritation of peripheral nerves. The older view that tendons, ligaments, and capsules of joints had a sparse nerve supply has been recently upset by Golgi and his followers, who have by the silver methods discovered numerous nerve endings in these structures. The main symptoms of general and traumatic neurasthenia are practically identical (Horsley), but great difficulty arises after accidents in determining whether local pains are in reality local or central. Since it is found that the painful spots (hysterogenic zones of Charcot) are identical in cases of neurasthenia of whatever origin, that the pain develops and persists often long after contusion has subsided, and that backache is effectually removed by massage, exercises and faradism of the erector spinæ muscles, Horsley has concluded that these pains are central in origin, and that counter irritation for their relief is useless.

Excepting the point of temperature in highly acute cases, no distinction can be drawn between the symptoms, course, prognosis, and treatment of traumatic neurasthenia and that produced by other causes. But we would urge on practitioners who are called to trivial sudden accidents, in the case of persons known to be mildly neurasthenic, the necessity of great caution in prognosis, and great care in keeping their patients in absolute rest of body and mind for a period of time which may seem to them and their friends superfluous and ridiculous.

[An excellent paper on the subject, by Mr. Victor Horsley, is to be found in the *Med. Soc. Trans.*, 1897. Vol. xx. p. 217.]

